# Predictors of abortions in Rural Ghana: a cross-sectional study

**DOI:** 10.1186/s12889-015-1572-1

**Published:** 2015-02-28

**Authors:** George Adjei, Yeetey Enuameh, Kwaku Poku Asante, Frank Baiden, Obed Ernest A Nettey, Sulemana Abubakari, Emmanuel Mahama, Stephaney Gyaase, Seth Owusu-Agyei

**Affiliations:** Kintampo Health Research Centre, P.O. Box 200, Kintampo, Ghana; PATH, Cantonments, P.O. Box CT 307, Accra, Ghana

**Keywords:** Spontaneous abortion, Induced abortion, Socio-demographic characteristics, Kintampo Health and Demographic Surveillance System, Sexual and Reproductive Health Survey

## Abstract

**Background:**

Abortion continues to be used as a method of family planning by many women. The complications of unsafe abortions are a major contributor to maternal mortality in sub-Saharan Africa, including Ghana. This study explored the influence of socio-demographic characteristics on abortions in 156 communities within the Kintampo Health and Demographic Surveillance System (KHDSS) area located in the middle part of Ghana.

**Methods:**

A survey on Sexual and Reproductive Health among a representative sample of females aged 15–49 years was conducted in 2011. They were asked about the outcome of pregnancies that occurred between January 2008 and December 2011. Data on their socio-demographic characteristics including household assets were accessed from the database of the KHDSS. Univariate and multivariate random effects logistic regression models were used to explore the predictors of all reported cases of abortion (induced or spontaneous) and cases of induced abortion respectively.

**Results:**

A total of 3554 women were interviewed. Of this total, 2197 women reported on the outcomes of 2723 pregnancies that occurred over the period. The number of all reported cases of abortions (induced and spontaneous) and induced abortions were 370 (13.6%) and 101 (3.7%) respectively.

Unmarried women were more likely to have abortion as compared to married women (aOR = 1.77, 95% CI [1.21-2.58], p = 0.003). Women aged 20–29 years were 43% less likely to have abortion in comparison with those within the ages 13–19 years (aOR = 0.57, 95% CI [0.34-0.95], p = 0.030). Women with primary, middle/junior high school (JHS) and at least secondary education had higher odds of having abortion as compared to women without education. Compared with the most poor women, wealthiest women were three-fold likely to have abortion.

Unmarried women had higher odds of having induced abortion as compared to married women (aOR = 7.73, 95% CI [2.79-21.44], p < 0.001). Women aged 20–29 years, 30–39 years and 40–49 years were less likely to have induced abortion as compared to those 13–19 years of age.

**Conclusion:**

Extra efforts are needed to ensure that family planning services, educational programs on abortion and abortion care reach the target groups identified in this study.

**Electronic supplementary material:**

The online version of this article (doi:10.1186/s12889-015-1572-1) contains supplementary material, which is available to authorized users.

## Background

Unsafe abortion is a major contributor to maternal mortality in sub-Saharan Africa [1-3]. According to the 2007 Ghana Maternal Health Survey (GMHS), unsafe abortion is the second largest cause of maternal mortality. About 15% of women in Ghana aged 15–49 years have had at least one abortion [3]. Unless concerted efforts are made to reduce unsafe abortion, Ghana will not be able to achieve the Millennium Development Goal (MDG) 5 of reducing maternal mortality by half by 2015. There is thus the need to investigate the factors that determine the resort to unsafe abortion in the country.

In many Ghanaian languages, abortion translated literally means “murder” and “spillage of blood” whereas miscarriage connotes a natural process [4,5]. Traditionally, abortion is a taboo subject among many tribes in Ghana. It is perceived as a shameful act that immoral women engage in [4-7]. This makes abortion a stigmatized undertaking in Ghanaian society [4].

Another factor that has contributed to the high incidence of unsafe abortions is the perception that all forms of abortion is illegal under current law in Ghana. The abortion law until 1985 stipulated that it is criminal for any woman to use any means to cause abortion [3,4]. The law was however amended in 1985 with the introduction of three exceptions which makes induced abortion partly legal. The three exceptions are when a pregnancy: (a) was as a result of rape, defilement or incest, (b) would pose a risk to the life of the pregnant woman or injury to her physical or mental health, (c) would lead to a substantial risk that if the child were born may lead to serious abnormality or disease [3,4].

Unsafe abortions in Ghana are noted to be significantly influenced by socio-demographic factors such as a number of children a woman already has, place of residence (rural or urban), marital status, religious affiliation, age, employment status and wealth [8-10]. However, most of the studies that explored the association of socio-demographic factors with abortion have been health facility based [10]. Findings from these studies may not be representative of the general population due to the influence of other factors that affect access to healthcare.

This study uses data from a large population-based cross-sectional study to explore the influence of socio-demographic background on the resort to abortions by women aged 15–49 years in the Kintampo North and South Districts in Ghana.

## Methods

### Study sites

The Kintampo North and South Districts in the Brong Ahafo Region of Ghana occupy a land area of 7,162 square kilometres. It is essentially rural and has population of about 143,000. The districts form the area covered by Kintampo Health and Demographic Surveillance System (KHDSS) that was established by Kintampo Health Research Centre (KHRC) as a core resource of a longitudinal follow-up of a defined population [11].

The prevalence of contraceptive use among women aged 15–49 years in the area was 30% in 2011 [12]. The total fertility rate (TFR) was also 4.0 in 2011 [12]. There are several health facilities in the study area that offer family planning services. Comprehensive post-abortion care is provided at the two districts hospitals in Kintampo North and Kintampo South districts respectively.

### Study design

Between July 2011 and December 2011, the KHRC conducted a Sexual and Reproductive Health (SRH) survey among females aged 15–49 years living in the 156 communities covered by the KHDSS. Using a questionnaire that contained close-ended questions, data were collected on reproductive health behaviour, contraception, fertility preferences, knowledge and prevalence of self-reported sexually transmitted infections. Three thousand five hundred and fifty four (3554) women were asked about the outcomes (i.e. live birth, stillbirth, spontaneous abortion, induced abortion) of any pregnancies they have ever had. To reduce recall errors, 2197 of the women with pregnancy outcomes from January 2008 to December 2011 were selected for this study.

The questionnaires were administered by well-trained field workers in the language that respondents were comfortable to speak in after pre-testing and revision. Because the study captured data on multiple pregnancy outcomes of each respondent, some of the respondents experienced both spontaneous and induced abortions. Evidence from studies has it that, induced abortions may be reported as spontaneous abortions by some respondents [13-15]. As a result of the afore-mentioned reasons, the study investigated two outcomes; induced abortion and all cases of abortion (induced or spontaneous) to explore their predictors. This was to ascertain whether any differences would be identified in predictors of the two outcomes.

Data on socio-demographic and household assets of women at the time close to each pregnancy outcome were extracted from the KHDSS database since those characteristics of the women varied over time.

### Data management and statistical analysis

The data were double entered into computer using the Visual fox pro software (version 9.0). Verification was applied to resolve discrepancies. The data were also checked for inconsistencies and outliers. The cleaned data were then exported to Stata (version 11.0) for statistical analysis.

The statistical analysis involved two parts. The first part was basic descriptive statistics where the means and percentages of individual characteristics were computed and tabulated. The second part involved exploratory univariate and multivariate random effects logistic regression with all cases of abortion (induced or spontaneous) and induced abortion as the main outcomes of interest. The variables that were significant in the univariate regression were included in the multivariate regression. The Wald adjusted test was then used to determine which variables should be significant in the multivariate regression. Clustering within respondents was adjusted for in the univariate and multivariate regressions since some of the women had multiple episodes of pregnancy outcomes.

Data on household asset of respondents were accessed from the database of the KHDSS and were used in Principal Component Analysis (PCA) to establish household wealth quintiles as explained by Filmer and Pritchett [16].

All statistical tests were two-tailed and a p-value of less than 0.05 was considered statistically significant.

The dataset used for the statistical analysis is fully available and can be accessed in Additional file 1.

### Ethical consideration

Ethical approval for the study was obtained from the KHRC Institutional Ethics Committee. All participants were individually consented for voluntary participation in the study. Parental consent and assent were obtained for minors (Respondents less than 18 years of age). Respondents who were unable to read or write had their thumb-printed consent forms countersigned by an impartial witness. To ensure respondents’ data confidentiality, respondents were identified with study codes only. Completed survey forms have been kept safely under lock and key at the KHRC.

## Results

### Demographic characteristics

A total of 3554 women were interviewed. Of this total, 2197 women reported on the outcomes of 2723 pregnancies that occurred over the period. The number of all reported cases of abortions (induced and spontaneous) and induced abortions were 370 (13.6%) and 101 (3.7%) respectively. The mean age at the time of the reported pregnancies was 28.6 years (Standard deviation = 7.2 years). More than half of the respondents were not married (53.1%) when they had their pregnancy outcome and about half (50.4%) of the women had no formal education when they experienced the pregnancy outcome. Table 1 presents the descriptive socio-demographic characteristics of respondents.

**Table 1:** **Distribution of socio-demographic responses**

**Socio-demographic variables**	**n (%)**
**Marital Status**	
Married	1278 (46.9)
Unmarried	1445 (53.1)
**Tota**l	**2723 (100.0)**
**Age**	
13-19	293 (10.8)
20-29	1293 (47.5)
30-39	931 (34.2)
40-49	206 (7.5)
**Total**	**2723 (100.0)**
**Educational level**	
No education	1305 (50.4)
Primary	479 (18.5)
Middle/JHS	650 (25.1)
Secondary+	154 (6.0)
**Total**	**2588 (100.0)**
**Household wealth**	
Most poor	610 (24.7)
More poor	543 (22.0)
Poor	509 (20.7)
Less poor	451 (18.3)
Wealthiest	352 (14.3)
**Total**	**2465 (100.0)**

### Socio-demographic predictors of all reported cases of abortions (induced or spontaneous)

Unmarried women were more likely to have abortion (either induced or spontaneous) as compared to married women (aOR = 1.77, 95% CI [1.21-2.58], p = 0.003) (Table 2). Women aged between 20 and 29 years were also less likely to have abortion as compared to the women within the ages of 13 and 19 years (aOR = 0.57, 95% CI [0.34-0.95], p = 0.030) (Table 2). The higher the level of education of the women, the more likely they were to have had abortion. Women with primary (aOR = 1.87, 95% CI [1.17-3.00], p = 0.009), middle/JHS (aOR = 2.06, 95% CI [1.33-3.20], p = 0.001) and at least secondary (aOR = 2.29, 95% CI [1.14-4.61], p = 0.020) education were also more likely to have abortion as compared to those without education (Table 2). The wealthiest women were also more likely to have had an abortion than the most poor women (aOR = 3.31, 95% CI [1.85-5.92], p < 0.001) (Table 2).

**Table 2:** **Socio-demographic factors associated with abortion (induced or spontaneous)**

**Variables**	**Univariate model OR (95% CI)**	**p**	**Multivariate model OR (95% CI)**	**p**
**Marital Status**				
Married	1		1	
Unmarried	2.08 (1.50-2.87)	<0.001	1.77 (1.21-2.58)	0.003
**Age**				
13-19	1		1	
20-29	0.48 (0.31-0.76)	0.002*	0.57 (0.34-0.95)	0.030^β^
30-39	0.43 (0.27-0.71)	0.001*	0.69 (0.39-1.21)	0.198^β^
40-49	0.62 (0.32-1.21)	0.160*	1.13 (0.54-2.38)	0.746^β^
**Educational level**				
No education	1		1	
Primary	2.03 (1.33-3.10)	0.001*	1.87 (1.17-3.00)	0.009*
Middle/JHS	2.60 (1.77-3.82)	<0.001*	2.06 (1.33-3.20)	0.001*
Secondary+	3.48 (1.882-6.426)	<0.001*	2.29 (1.14-4.61)	0.020*
**Household wealth**				
Most poor	1		1	
More poor	1.11 (0.65-1.91)	0.690*	1.12 (0.66-1.91)	0.684*
Poor	2.11 (1.26-3.55)	0.005*	1.49 (0.88-2.54)	0.137*
Less poor	2.12 (1.25-3.60)	0.005*	1.56 (0.91-2.69)	0.110*
Wealthiest	4.78 (2.72-8.40)	<0.001*	3.31 (1.85-5.92)	<0.001*

*Wald adjusted p < 0.01 ρ for multivariate regression = 0.424.

^β^Wald adjusted p = 0.0425.

### Socio-demographic predictors of only reported cases of induced abortion

Unmarried women had a higher odds of having had an induced abortion than married women (aOR = 7.73, 95% CI [2.79-21.44], p < 0.001) (Table 3). Compared with women between 13 and 19 years of age, women aged between 20 and 29 years (aOR = 0.26, 95% CI [0.12–0.60], p = 0.002), 30 and 39 years (aOR = 0.19, 95% CI [0.06-0.54], p = 0.002) and 40 to 49 years (aOR = 0.12, 95% CI [0.02-0.82], p = 0.031) were less likely to have had induced abortion (Table 3). The higher the level of education of the women, the more likely for them to have had induced abortion. However, this relationship was not significant (Wald-adjusted p = 0.051) (Table 3). Household wealth (Wald-adjusted p = 0.082) of women was found not to have significant influence on whether they had induced abortion or not (Table 3).

**Table 3:** **Socio-demographic factors associated with induced abortion**

**Variables**	**Univariate model OR (95% CI)**	**P**	**Multivariate model OR (95% CI)**	**p**
**Marital Status**				
Married	1		1	
Unmarried	14.26 (1.04-2.72)	<0.001	7.73 (2.790-21.440)	<0.001
**Age**				
13-19	1		1	
20-29	0.17 (0.06-0.49)	0.001*	0.26 (0.12-0.60)	0.002*
30-39	0.05 (0.01-0.20)	<0.001*	0.19 (0.06-0.54)	0.002*
40-49	0.03 (0.00-0.29)	0.002*	0.12 (0.02-0.82)	0.031*
**Educational level**				
No education	1		1	
Primary	5.49 (2.04-14.73)	0.001*	2.61 (1.027-6.630)	0.044^φ^
Middle/JHS	6.39 (2.44-16.74)	<0.001*	2.40 (0.994-5.773)	0.051^φ^
Secondary+	16.92 (3.66-78.23)	<0.001*	5.51 (1.523-19.963)	0.009^φ^
**Household wealth**				
Most poor	1		1	
More poor	0.89 (0.28-2.87)	0.842*	1.04 (0.34-3.21)	0.950^θ^
Poor	2.47 (0.83-7.37)	0.104*	1.49 (0.51-4.39)	0.470^θ^
Less poor	2.61 (0.88-7.77)	0.084*	1.85 (0.64-5.33)	0.255^θ^
Wealthiest	6.90 (2.21-21.58)	0.001*	4.02 (1.29-12.54)	0.017^θ^

*Wald adjusted p < 0.01 ρ for multivariate regression = 0.591.

^φ^Wald adjusted p = 0.051 ^θ^Wald adjusted p = 0.082.

## Discussion

Women of reproductive age seeking unsafe abortion clandestinely are known to be driven by forces of stigmatization, unwanted pregnancy, ignorance of the abortion law, low and failure in contraceptive use [4,8,9,17-19]. However, this study investigated other factors that serve as a medium for these driving forces to thrive. The outcomes of interest in the study were induced abortion alone and all cases of reported abortion (combination of induced and spontaneous abortion). In addition, respondents of the study were women aged 15–49 years but some of them were 13 years of age when they had their pregnancy outcomes in the past.

In this study we found that, women who were not married were more likely to have all cases of abortions (induced or spontaneous) as compared to those who were married. Findings from the 2008 Ghana Demographic and Health Survey indicates that contraceptive use among married women has almost doubled over the past 20 years [20]. This reason may account for the low odds of married women having unsafe abortion. Also, studies carried out by Ahiadeke et al. [10] and Mote et al. [9] in different districts of Ghana and others from sub-Saharan Africa had results that were in line with this finding [9,10,13,21,22]. Stigmatization associated with out-of-wedlock pregnancies in Ghana [23] and perceptions of it being dishonourable in Burkina Faso [13] could be contributing factors to the practice of unsafe abortions. Some unmarried women wanting to postpone childbearing until marriage, others not having adequate financial support to cater for their unborn child and problems associated with informal relationships have been advanced as possible reasons for unsafe induced abortions [17,23,24].

In addition, the findings of this study suggest that the higher the educational level of women, the more likely they are to have abortion (induced or spontaneous). This pattern was observed in the findings of the Mote, Ahiadeke et al. studies and Ghana Maternal Health Survey (GMHS) [3,8-10]. According to Sundaram et al. study (2012), the possible explanation could be that better educated women are more likely to have greater access to information through media and may also have better knowledge of the abortion law [8]. However, this result is in contrast with the finding of a study which was conducted in Ethiopia in which women with higher education were not likely to have induced abortion [25]. The women aged 20–29 years were also found to have lower odds of having abortion as compared to adolescents (13–19 years). Several other studies have reported findings similar to this [13,21,22,26]. The probable explanation for this finding is that women aged 13–19 years are mostly under strict parental (or guardian) control and therefore resort to unsafe abortions for fear of being disowned by their parents [13,23]. A study conducted in Burkina Faso reported that women under parental control were seven-fold as likely to have induced abortion when compared with those not under such control [13]. Moreover, a significant proportion of adolescent may have less access to financial resources to pay for safe induced abortion. Adolescents may also be less likely to know where to get abortion and may be more likely influenced by stigma as compared to older women [23]. According to the Ghana Maternal Health Survey, lack of money to cater for babies is one of the major reasons cited by women who had induced abortion [3]. This suggests that adolescents may use induced abortions as a family planning option for unplanned pregnancy; an indicator of unmet need for contraception.

Wealthiest women in this study were found to have higher likelihood of having all cases of abortions (induced or spontaneous) as compared to the most poor women. This is in line with the fact that odds of having induced abortion in Ghana is 67%-80% higher among women in the top two wealth quintiles than among the those of the lowest quintile [23]. The findings from other studies [27-30] have also shown that poor women suffer more from unsafe induced abortion than wealthiest women. Wealthiest women are financially empowered and can afford to have safe induced abortions in better health facilities as compared to poor women [27,31]. Perhaps these findings explain the increased likelihood of the wealthiest women to have induced abortion.

The marital status of women was found to be significantly associated with induced abortion in this study. This finding is consistent with the influence of marital status on all reported cases of abortion (induced or spontaneous) and findings of several other studies [9,10,13,21,22]. In addition, it was observed in the current study that the higher the educational level of the women, the more likely for them to have induced abortion. This finding is similar to those who had all reported cases of abortions (induced or spontaneous) and findings of other studies [3,8-10] but not statistically significant. Also, the wealthiest women were found in this current study to be more likely to have induced abortion than the most poor women. This finding is in line with the finding of the association of household wealth with all reported cases of abortions (induced or spontaneous) and a study conducted in Ghana [8]. However, the relationship between household wealth and induced abortions was not statistically significant.

According to the 2008 Ghana Demographic and Health Survey (GDHS), Total Fertility Rate (TFR) in rural Ghana declined from 5.6 births per woman to 4.9 births per woman within a period of 5 years. However the change in contraceptive use in rural areas over that same period was not captured by the report(13). That information gap leads to possible inference of induced abortion being a major contributor to the declining TFR in rural Ghana. Concerted efforts should therefore be made to reduce unsafe abortions. Reduction in unsafe abortions could be done by: (a) making contraceptives affordable and easily accessible (b)improving access to induced abortions through intensified educational programs on the current abortion law and its provisions, and (c) improving the rights of persons requiring abortion services in Ghana.

This study however has few limitations. The status of birth outcomes were self-reported and as such it is possible that respondents reported a lot more induced abortions as spontaneous.. The respondents may also find it difficult to accurately recall some of the past pregnancy outcomes. The study could not also ascertain from respondents whether the induced abortions done were perceived to be safe or not. The study also has a limitation of being cross-sectional, as causal relationships could not be established between the identified factors and abortions.

## Conclusion

The findings of this study have shown that younger and unmarried women experience more unwanted pregnancies that leads to induced abortions or all reported cases of abortions (induced or spontaneous). However the status of women being wealthiest or more educated was found to be a correlate of only all reported cases of abortions (induced or spontaneous). These findings points to the need for extra efforts to ensure that family planning services, educational programs on abortion and abortion care reach target groups identified in this study.

## Additional file

Additional file 1:
**STATA dataset used for analysis.**
